# Supplementary material for: Childhood factors associated with suicidal ideation among South African youth: A 28-year longitudinal study of the Birth to Twenty Plus cohort
Source: PLoS Med. 2022 Mar 15;19(3):e1003946. doi: 10.1371/journal.pmed.1003946 (PMC8923476; doi:10.1371/journal.pmed.1003946)
Supplement: S1 Appendix — (PDF) [file pmed.1003946.s002.pdf]

Suicide ideation

Age 14

	**Not true**	**Sometimes true**	**True**	**Very true**
I think about killing myself				

Age 17 and age 22

**Have you recently,**	**Definitely not**	**I don't think so**	**Has crossed my mind**	**Definitely have**
Found that the idea of taking your own life kept coming into your mind?				

Age 28

	**No**	**Yes**
In the past month, has the thought of ending your life been on your mind?		

Pitt Inventory (6 months)

	**Yes**	**No**	**Don't know**
Do you sleep well?			
Do you easily lose your temper?			
Are you worried about your looks?			
Have you a good appetite?			
Are you as happy as you ought to be?			
Do you easily forget things?			
Have you as much interest in sex as ever?			
Is everything a great effort?			
Do you feel ashamed for any reason?			
Can you relax easily?			
Can you feel the baby is really yours?			
Do you want someone with you all the time?			
Are you easily woken up?			
Do you feel calm most of the time?			
Do you feel that you are in good health?			
Does food interest you less than it did?			
Do you cry easily?			
Is your memory as good as ever?			
Have you less desire for sex than usual?			
Have you enough energy?			
Are you satisfied with the way you are coping with things?			
Do you worry a lot about the baby?			
Do you feel unlike your normal self?			
Do you have confidence in yourself?			

South African Child Assessment Schedule (ages 5, 7 and 10)

I have some questions I would like to ask you about problems and skills some children have. I would like you to tell me the extent to which it is true that your child has these. In responding tell me whether it is –

0 = Not True 1 = Sometimes True 2 = Often True

0	1	2	1. Does _____ seem fragile or cry when an adult just looks at her
0	1	2	2. Does _____ accept and listen to criticism calmly (hri 46gut)
0	1	2	3. Does _____ accept restrictions from adults (hri 42rule)
0	1	2	4. Does _____ act too young for his / her age (a5att)
0	1	2	5. Does _____ adjust well to changes in the classroom routine (44gut)
0	1	2	6. Is ________ affectionate towards other (hri 22peer)
0	1	2	7. Is ________ an angry child
0	1	2	8. Does _____ approach new experiences confidently, without fear (a 11ag)
0	1	2	9. Does _____ argue (a 11ag)
0	1	2	10. Does _____ avoid activities which s/he is not good at
0	1	2	11. Does _____ brag or boast (a 19ag)
0	1	2	12. Does _____ bully or is s/he mean to others (a 19ag)
0	1	2	13. Is ________ unable to concentrate, pay attention for long (a 20att)
0	1	2	14. Is ________ unable to get his / her mind off certain thoughts (thtdis)
0	1	2	15. Is ________ unable to sit still, does squirm (a 23att)
0	1	2	16. Can ______ accept things not going his / her way (hri 12gut)
0	1	2	17. Does _____ carry out requests and directions responsibly (h 32rule)
0	1	2	18. Does _____ cling to adults, is too dependent? (c 11soc)
0	1	2	19. Is ________ co-operative?
0	1	2	20. Does _____ complain of aches or pains in arms or legs (a 29sc)
0	1	2	21. Does _____ complain of dizziness (a 20sc)
0	1	2	22. Does _____ complain of headaches (a 31sc)
0	1	2	23. Does _____ complain of loneliness (a 32ad)
0	1	2	24. Does _____ complain of nausea or feeling sick (a 33sc)
0	1	2	25. Does _____ complain of stomach aches or cramps (a 34sc)
0	1	2	26. Does _____ complete homework (hri 26gdst)
0	1	2	27. Is ________ confused (does not attend to what is going on) (a 35att)

0	1	2	28. Does _____ cry without good reason (a 38ad)
0	1	2	29. Is ________ curious or enthusiastic about new activities (schread)
0	1	2	30. Does _____ daydream or get lost in his / her thoughts (a 40 att)
0	1	2	31. Does _____ defy authority or break rules (oppos)
0	1	2	32. Does _____ deliberately destroy things that belong to others
0	1	2	33. Does _____ demand attention (a 47ag)
0	1	2	34. Does _____ destroy his own things (a 48ag)
0	1	2	35. Is ________ disobedient at home (a 50ag)
0	1	2	36. Is ________ disobedient at school (a 51ag)
0	1	2	37. Does _____ do unusual original, creative work (hri 11gdst)
0	1	2	38. Does _____ eat poorly (c 25soc)
0	1	2	39. Is ________ easily jealous (a 61ag)
0	1	2	40. Does _____ express needs and feelings appropriately (hri 45rule)
0	1	2	41. Does _____ face the pressures of competition well (hri 38gut)
0	1	2	42. Does _____ have specific fears? (ad)
0	1	2	43. Does _____ fear s/he might do something bad (a 69ad)
0	1	2	44. Does _____ feel good about him / herself (hri 2gut)
0	1	2	45. Does _____ feel s/he has to be perfect (a 72ad)
0	1	2	46. Does _____ feel too guilty (a 74ad)
0	1	2	47. Does _____ feel worthless or inferior (a 75ad)
0	1	2	48. Does _____ feel / complain that no one loves him / her (a 73ad)
0	1	2	49. Does _____ follow rules and directions (hri 18rule)
0	1	2	50. Does _____ function well even with distractions (hri 1gdst)
0	1	2	51. Is ________ generally relaxed (hri 23gut)
0	1	2	52. Does _____ get teased by other children? (c 38soc)
0	1	2	53. Is ________ good at counting (maths)? (hri 35 gdst)
0	1	2	54. Is ________ happy (hri 10peer)
0	1	2	55. Is ________ hard to understand what is saying (schready)
0	1	2	56. Does _____ have a good sense of humour, smile a lot (hri 4peer)
0	1	2	57. Does _____ have many friends (hri 39peer)
0	1	2	58. Does _____ have strange ideas – if TRUE describe

____________________________________ (c 85thtdis)

0	1	2	59. Does _____ hear things that aren't there – if TRUE describe

_____________________________________ (c 40thtdis)

0	1	2	60. Does _____ hesitate to try new things
0	1	2	61. Is ________ impulsive or does act without thinking (hri 5gdst)
0	1	2	62. Is ________ independent, does like to do things without help
0	1	2	63. Is ________ interested in school work (hri 5gdst)
0	1	2	64. Is ________ irritable? (a 96ag)
0	1	2	65. Is ________ a good reader for his / her grade? (hri 47gdst)
0	1	2	66. Does _____ know his / her strengths and weaknesses? (hri 43peer)
0	1	2	67. Does _____ look unhappy without good reason? (a 102ad/ws)
0	1	2	68. Is ________ loud, noisy? (a 105ag)
0	1	2	69. Is ________ loving, shows affection to others
0	1	2	70. Is ________ 's mood even and stable? (hri 15gut)
0	1	2	71. Is ________ nervous movements or twitching? (a 109att)
0	1	2	72. Is ________ nervous, high strung or tense? (a 110ad)
0	1	2	73. Is ________ able to take turns and share? (schready)
0	1	2	74. Is ________ not liked by other children? (c 48soc)
0	1	2	75. Is ________ overactive, restless, unable to sit still
0	1	2	76. Is ________ overtired, sleepy during the day (a 116sc, schready)
0	1	2	77. Is ________ overweight? (c 55soc)
0	1	2	78. Does _____ physically attack people? (a 120ag)
0	1	2	79. Does _____ play enthusiastically? (hri 25peer)
0	1	2	80. Is ________ polite and courteous? (hri 54rule)
0	1	2	81. Is ________ poor at school work? (a 125att)
0	1	2	82. Is ________ poorly co-ordinated or clumsy? (a 126att.c 62soc)
0	1	2	83. Does _____ prefer playing with younger children? (c 64soc)
0	1	2	84. Does _____ prefer to be alone? (a 127ws)
0	1	2	85. Does _____ have problems with eyes not corrected by glasses
0	1	2	86. Does _____ require restrictions to control him / her? (hri 53rule)
0	1	2	87. Does _____ have rashes or other skin problems (a 133sc)
0	1	2	88. Does _____ refuse to talk in certain situations? (a 135 ws)
0	1	2	89. Does _____ repeat certain acts over and over? (c 66thtdis)

0	1	2	90. Does _____ resolve peer problems on his / her own? (hri 16gut)
0	1	2	91. Is ________ sad or depressed? (a 141ad/ws)
0	1	2	92. Does _____ scream? (a 144ag)
0	1	2	93. Is ________ secretive, does keep things to self? (a 145ws)
0	1	2	94. Does _____ seem to think that others are out to get him / her (-ad)
0	1	2	95. Does _____ see things that aren't there? (c 70thtdis)
0	1	2	96. Is ________ self-conscious or easily embarrassed? (a 150ad)
0	1	2	97. Is ________ a self-starter, begins without waiting for adults? (gdst)
0	1	2	98. Does _____ share things with others? (hri 6peer)
0	1	2	99. Does _____ show interest in people around him / her? (hri 17peer)
0	1	2	100. Does _____ show off or clown? (a 160ws)
0	1	2	101. Is ________shy or timid?(a 160ws)
0	1	2	102. Does _____ stare blankly? (c 80thtdis)
0	1	2	103. Does _____ stare into space or seem preoccupied?(a 168att/ws)
0	1	2	104. Does _____ start fights? (a 169ag)
0	1	2	105. Does _____ have strange behaviour

Describe ____

0	1	2	106. Is ________stubborn, sullen or irritable (c86 ws/a 177ag)
0	1	2	107. Does _____ have sudden changes in mood or feelings? (a 179ag)
0	1	2	108. Does _____ pull a face, sulk or pout? (a 180ws)
0	1	2	109. Is ________suspicious of others? (a 181ad)
0	1	2	110. Does _____ talk too much? (a 186ag)
0	1	2	111. Does _____ tease other kids? (a 187ag)
0	1	2	112. Does _____ have temper tantrums or hot temper? (a 188ag)
0	1	2	113. Does _____ threaten people? (a 190ag)
0	1	2	114. Is ________too fearful or anxious? (a 193ad)
0	1	2	115. Does _____ try to help others? (a 193ad)
0	1	2	116. Is ________trustworthy? (hri 29rule)
0	1	2	117. Is ________underactive, slow-moving or lacks energy? (a 196ws)
0	1	2	118. Does _____ vomit, throw up? (a 203sc)
0	1	2	119. Is ________well liked by other children his / her age? (hri 34peer)
0	1	2	120. Is ________well-behaved in school? (hri 7rule)

0	1	2	121. Is ________withdrawn, doesn't get involved with others? (a 210ws)
0	1	2	122. Does _____ work up to potential (hri 40 gdst)
0	1	2	123. Does _____ work well without adult support? (hri 30gdst)
0	1	2	124. Does _____ suck his / her thumb?
0	1	2	125. Does _____ do things to hurt him / herself (e.g. bang head on wall)
0	1	2	126. Does _____ worry (a 212ad)
0	1	2	127. Does _____ not feel sorry after misbehaving (cbcl 16del)
0	1	2	128. Does _____ lie or cheat (cncl 81del)
0	1	2	129. Does _____ steal (cbcl 81del)
0	1	2	130. Does _____ swear or use obscene language (cbcl 90del)
0	1	2	131. Does _____ wet the bed at night (cbcl 90del)
0	1	2	132. Does _____ 's have close friends who are naughty? (cbcl 81del)
0	1	2	133. Does _____ smoke or use drugs (cbcl 105del)
0	1	2	134. Does _____ skip school (cbcl 101 del)
0	1	2	135. Does _____ set fires (cbcl 81del)

Adverse childhood experiences (ages 5, 7, 11, and 13)

**Thinking back to your childhood, the first 18 years of your life, please tick each one that applies to you**			
**ACE**	**Question**	**Yes**	**No**
**Physical abuse**	Did a parent or other adult in the household **often or very often**… Push, grab, slap, or throw something at you? **OR** **Ever** hit you so hard that you had marks or were injured?		
**Sexual abuse**	Did an adult or person at least 5 years older than you **ever**… Touch or fondle you or have you touch their body in a sexual way? **OR** Attempt or actually have oral, anal, or vaginal intercourse with you?		
**Emotional abuse**	Did a parent or other adult in the household **often or very often**… Swear at you, insult you, put you down, or humiliate you? **OR** Act in a way that made you afraid that you might be physically hurt?		
**Emotional neglect**	Did you **often or very often** feel that …		

	No one in your family loved you or thought you were important or special?<br>**OR**<br>Your family didn't look out for each other, feel close to each other, or support each other?		
**Physical neglect**	Did you **often or very often** feel that …<br>You didn't have enough to eat, had to wear dirty clothes, and had no one to protect you?<br>**OR**<br>Your parents were too drunk or high to take care of you or take you to the doctor if you needed it?		
**Parental divorce**	Were your parents **ever** separated or divorced?		
**Witnessing domestic violence**	Was your mother or stepmother:<br>**Often or very often** pushed, grabbed, slapped, or had something thrown at her?<br>**OR**<br>**Sometimes, often, or very often** kicked, bitten, hit with a fist, or hit with something hard?<br>**OR**<br>**Ever** repeatedly hit at least a few minutes or threatened with a gun or knife?		
**Alcohol and/or drug abuse in the household**	Did you live with anyone who was a problem drinker or alcoholic or who used street drugs?		
**Mental illness in the household**	Was a household member depressed or mentally ill, or did a household member attempt suicide?		
**Legal trouble in the household**	Did a household member go to prison?		
**Chronic illness (other than mental illness) in the household**	Was there anyone in your household who was chronically ill when you were a child?		
**Unemployment of parent/caregiver**	Was one or more of your parents/caregivers mostly unemployed during your childhood because they could not get a job?		
**Death of parent/caregiver**	Did either of your parents/caregivers pass away before you turned 18?		
