# Supplementary material for: Childhood factors associated with suicidal ideation among South African youth: A 28-year longitudinal study of the Birth to Twenty Plus cohort
Source: PLoS Med. 2022 Mar 15;19(3):e1003946. doi: 10.1371/journal.pmed.1003946 (PMC8923476; doi:10.1371/journal.pmed.1003946)
Supplement: S1 Table — (DOCX) [file pmed.1003946.s003.docx]

**S1 Table.** Comparison of the characteristics of participants included and nonincluded in the analysis sample

|  | Included participants  (N=2,020) | Nonincluded participants  (N=1,253) | *P* |
| --- | --- | --- | --- |
| Birth weight (Kg), mean (SD) | 3.06 (0.51) | 3.08 (0.52) | 0.166 |
| Child male sex, n (%) | 1042 (51.6) | 637 (50.8) | 0.705 |
| Low maternal age, n (%) | 149 (7.4) | 52 (4.2) | <0.001 |
| Low maternal education, n (%) | 1096 (59.1) | 615 (57.0) | 0.271 |
| Household crowding, n (%) | 772 (44.6) | 249 (27.1) | <0.001 |
| Poverty, n (%) | 890 (44.1) | 598 (47.7) | 0.044 |
| Material deprivation, mean (SD) | 0.15 (0.92) | -0.62 (1.07) | <0.001 |
| Loss, mean (SD) | 0.07 (0.99) | -0.30 (0.99) | <0.001 |
| Family dynamics, mean (SD) | 0.12 (1.00) | -0.49 (0.81) | <0.001 |
| Abuse and violence, mean (SD) | 0.00 (1.01) | 0.04 (0.91) | 0.762 |
| Postnatal maternal depression, mean (SD) | 199 (17.2) | 127 (17.8) | 0.785 |
| Birth order, mean (SD) | 2.11 (1.08) | 2.15 (1.06) | 0.283 |
| Parity, mean (SD) | 2.18 (1.23) | 2.22 (1.20) | 0.385 |
| Previous abortions/stillbirths, n (%) | 219 (10.8) | 166 (13.2) | 0.043 |
| Externalising problems, mean (SD) | 0.01 (0.98) | -0.07 (1.07) | 0.166 |
| Internalising problems, mean (SD) | 0.08 (0.97) | -0.41 (1.07) | <0.001 |

SD, Standard Deviation
