# Supplementary material for: Childhood factors associated with suicidal ideation among South African youth: A 28-year longitudinal study of the Birth to Twenty Plus cohort
Source: PLoS Med. 2022 Mar 15;19(3):e1003946. doi: 10.1371/journal.pmed.1003946 (PMC8923476; doi:10.1371/journal.pmed.1003946)
Supplement: S2 Table — (DOCX) [file pmed.1003946.s004.docx]

**S2 Table.** Count and proportion of missing data in the analysis variable

|  | n (%) |
| --- | --- |
| Birth weight (Kg), mean (SD) | 4 (0.2) |
| Child male sex, n (%) | 0 |
| Low maternal age, n (%) | 2 (0.1) |
| Low maternal education, n (%) | 167 (8.3) |
| Household crowding, n (%) | 290 (14.4) |
| Poverty, n (%) | 0 |
| Material deprivation, mean (SD) | 138 (6.83) |
| Loss, mean (SD) | 140 (6.93) |
| Family dynamics, mean (SD) | 158 (7.82) |
| Abuse and violence, mean (SD) | 897 (44.4) |
| Postnatal maternal depression, mean (SD) | 865 (42.8) |
| Birth order, mean (SD) | 0 |
| Parity, mean (SD) | 0 |
| Previous abortions/stillbirths, n (%) | 0 |
| Externalising problems, mean (SD) | 399 (19.7) |
| Internalising problems, mean (SD) | 398 (19.7) |

SD, Standard Deviation
