# Supplementary material for: Childhood factors associated with suicidal ideation among South African youth: A 28-year longitudinal study of the Birth to Twenty Plus cohort
Source: PLoS Med. 2022 Mar 15;19(3):e1003946. doi: 10.1371/journal.pmed.1003946 (PMC8923476; doi:10.1371/journal.pmed.1003946)
Supplement: S3 Table — (DOCX) [file pmed.1003946.s005.docx]

**Table S3.** Multivariable associations between childhood factors and suicidal ideation using factors associated with suicidal ideation in the univariable analysis at *P* < 0.05

|  | OR (CI) | *P* |
| --- | --- | --- |
| **Males** |  |  |
| Socioeconomic adversity score | 1.13 (1.02-1.25) | 0.026 |
| Birth order (trend) | 1.02 (0.93-1.12) | 0.678 |
| Externalising problems | 1.22 (1.08-1.39) | 0.001 |
| Internalising problems | 1.11 (0.98-1.26) | 0.093 |
|  |  |  |
| **Females** |  |  |
| Low birth weight | 1.33 (1.05-1.68) | 0.044 |
| ACE score | 1.11 (1.01-1.21) | 0.023 |
| Birth order (trend) | 1.14 (1.06-1.23) | < 0.001 |
| Externalising problems | 1.16 (1.03-1.31) | 0.010 |

ACE, Adverse Childhood Experiences
