# Supplementary material for: Childhood factors associated with suicidal ideation among South African youth: A 28-year longitudinal study of the Birth to Twenty Plus cohort
Source: PLoS Med. 2022 Mar 15;19(3):e1003946. doi: 10.1371/journal.pmed.1003946 (PMC8923476; doi:10.1371/journal.pmed.1003946)
Supplement: S1 Fig — Bt20+, Birth to Twenty Plus. (DOCX) [file pmed.1003946.s006.docx]

**S1 Fig.** Representation of the Birth to Twenty Plus cohort assessments used in this investigation


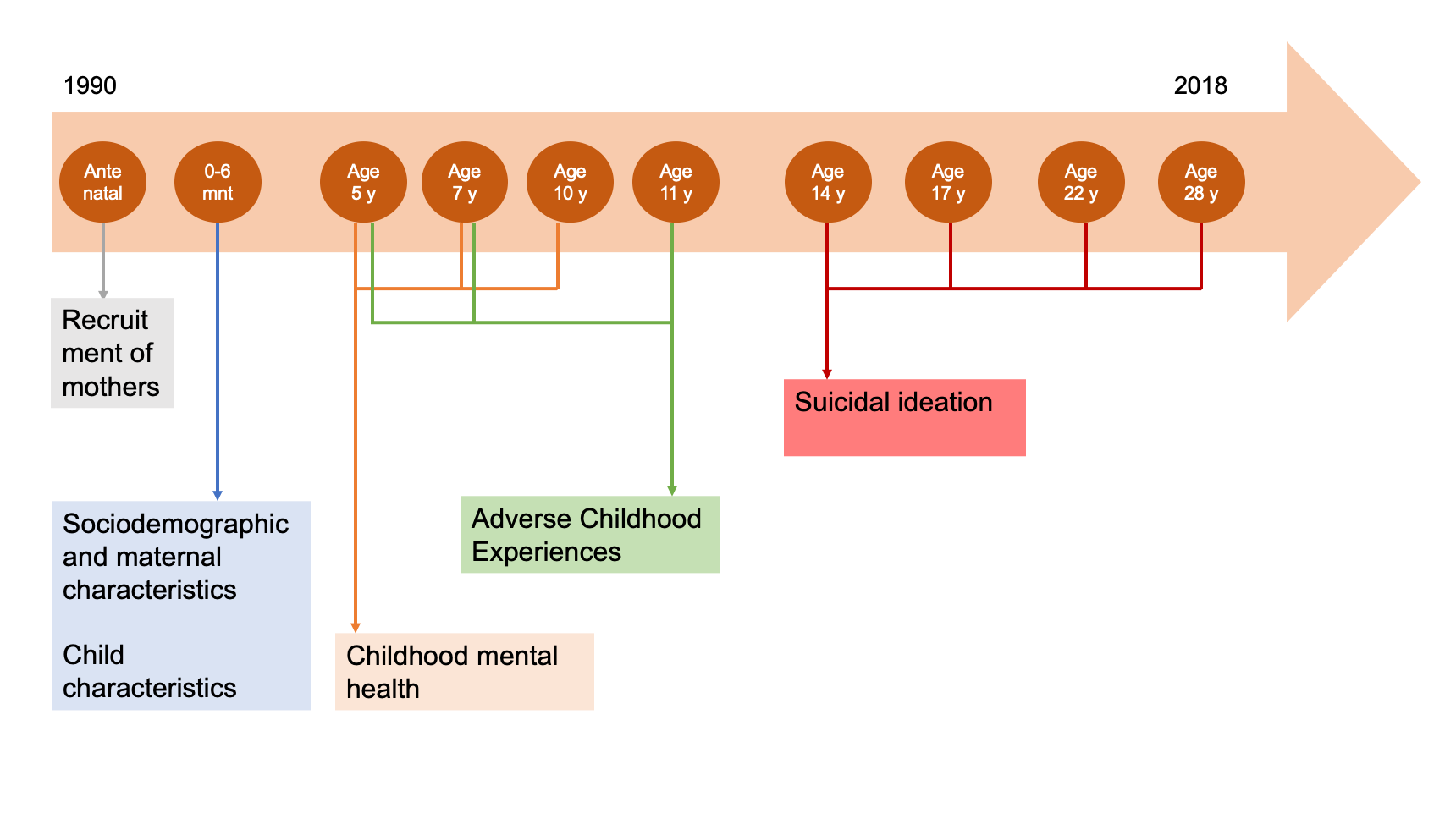


The figure represents the ages of the participants (in months [mts] and years [y]) during which the variables used in the study were assessed.
