# Supplementary material for: Childhood factors associated with suicidal ideation among South African youth: A 28-year longitudinal study of the Birth to Twenty Plus cohort
Source: PLoS Med. 2022 Mar 15;19(3):e1003946. doi: 10.1371/journal.pmed.1003946 (PMC8923476; doi:10.1371/journal.pmed.1003946)
Supplement: Attachment — Submitted filename: 2022-01-06_R1_Response Letter.docx [file pmed.1003946.s007.docx]

**Comments from the Academic Editor**

**Comment 1**. The authors have selectively focused on adversity and not on potentially protective or mitigating factors e.g. relating to schooling/educational success, social capital/extended family indicators (if measured). This appears to be a limitation.
**Response 1**. We agree on this limitation. The aim of this paper was to identify risk factors for suicidal ideation, and the choice of our variables mostly reflects this perspective (see also our reply to Comment 2 for our conceptual model). Although some variables may represent risk or protective factors depending on the category used as reference (e.g., the OR for low maternal education *vs* non-low maternal education in males is 1.31, meaning that non-low maternal education has a protective effect: 1/1.31 = OR 0.78), we agree that more research is needed to identify specific protecting and mitigating factors following established conceptual models.

To take this comment into account, we added the following limitation to the discussion (page 17, lines 437-441 ***in document with tracked changes***):

Finally, the childhood factors investigated in our study reflect a conceptual model that mostly emphasizes risk factors rather than protective and mitigating factors. To enhance suicide prevention, further research is needed to understand the factors decreasing and buffering suicide risk, especially for children exposed to socioeconomic adversity and those exhibiting behavioral problems.

**Comment 2**. Was there any conceptual model that drove the selection the individual, familial and environmental exposures? for example, was substance use considered as a factor?

**Response 2.** We have followed the developmental model of suicide proposed by Turecki and Brent (*Lancet*, 2016). This has now been clarified in the introduction as follows (page 5, lines 131-136):

Following Turecki and Brent’s developmental model of suicide risk [1], a wide range of potential childhood risk factors were investigated, including early-life socioeconomic adversity, ACEs, and children’s externalizing and internalizing problems. These factors – considered in the model as predisposing and developmental, as opposed to precipitating, factors – were hypothesized to increase vulnerability to suicide during the life-course and may be targeted by population-based suicide prevention strategies.

**Comment 3**. Is it reasonable to expect that predictors of suicidal ideation at those different time-points would be the same?
**Response 3.** Thank you for this comment and interesting observation. While the literature suggests the existence of some age-specific risk factors for suicide-related outcomes (e.g., puberty timing), no clear differences have been found for the risk factors including those investigated in our study. For example, in a previous meta-analysis on the early-life origins of suicide, there was no evidence of differential associations of early-life factors (in-utero/perinatal characteristics, family, and parental socioeconomic characteristics at childbirth) on suicide risk in youth vs adults (Orri et al. *Lancet Psychiatry* 2019). In a Canadian longitudinal study investigating internalizing and externalizing problems as risk factors for suicide-related outcomes from age 13 to 20 years, associations were similar across the investigated period (Orri et al. *Pediatrics* 2020). Therefore, there was little evidence to hypothesize that the specific risk factors that we investigated have different associations for suicidal ideation at different time-points. It is also important to note that we decided to focus on lifetime suicidal ideation to have sufficient statistical power to reliably test our hypotheses.

**Comment 4**. It is surprising that postnatal depression is not a predictor. What could be the reason for that?

**Comment 4.** Thank you for this comment. In our previous studies using the Birth to Twenty Plus cohort (Orri et al. 2021 *JAMA Netw Open*; Verkuijl et al., *Lancet Psych* 2014), we found associations between postnatal depression and offspring depression later in life, suggesting that maternal depression is associated with child internalizing problems. The absence of associations with suicidal ideation in the present study may indicate that, although maternal depression increases risk for offspring depression, it is not sufficient to differentiate participants who consider suicide (which is often an extreme outcome of depression) from those who do not. According to this and previous studies (Orri et al, 2018 *Lancet Psych*; Commisso et al. *Psych Med* 2021) and models (Turecki and Brent, *Lancet* 2016), suicide risk is influenced by the presence of both factors influencing mood disorders (such as maternal depression) and other factors such as externalizing problems and personality traits (e.g., impulsivity, behavioral disinhibition) that jointly increase suicide risk.

However, it is also important to acknowledge that associations found in samples from HICs do not necessarily hold in LMICs, where the social, economic, and adversity context is substantially different from that in HICs. This call for caution in interpreting the findings from studies in LMICs with the lens of our knowledge from HICs and, in our view, further justifies the importance and originality of our study.

To address this comment, we have now added the following in the Discussion section (pages 14-15, lines 365-371):

Finally, different from studies in HICs [55-56], we did not find an association between maternal postnatal depression and offspring suicidal ideation. However, previous Bt20+ studies have found that maternal postnatal depression is associated with offspring internalizing problems in both childhood [40] and early adulthood [57]. Therefore, the absence of an association with suicidal ideation in the present study may indicate that although maternal depression increases risk for offspring internalizing problems, it is not sufficient to differentiate participants who consider suicide from those who do not in the specific socioeconomic context of South Africa.

**Comment 5**. I agree with the statistical reviewer's concerns about the way that variables were selected for inclusion into the multivariable analysis, and also the interpretation of interactions.
**Response 5.** To address this comment, we have now modified the way in which the variables were selected for inclusion into the multivariable model as well as the interpretation of the interactions. Please, refer to Response 6 of R#1 for the details.

Other editorial points:

**Comment 6**. Data availability statement: Thank you for providing the link to request data access. Please clarify if there is a more direct link available (e.g. <https://www.wits.ac.za/coe-human/open-access-datasets/>). If possible please provide a (non-author) contact email address to which data inquiries can be directed.

According to this website, the following text should be included in the acknowledgements: “The support of the DSI-NRF Centre of Excellence in Human Development at the University of the Witwatersrand, Johannesburg in the Republic of South Africa towards this research is hereby acknowledged. Opinions expressed and conclusions arrived at, are those of the author and are not to be attributed to the CoE in Human Development.” It seems only part of this statement was included.
**Response 6.** Access to all data managed by the COE in Human Development is processed through this link. We have included the study’s principal investigator (a non-author) who can be contacted for access to the data. The data availability statement has been amended as follows to reflect this (page 20, lines 504-507):

Bt20+ is housed in the DSI-NRF Centre of Excellence in Human Development at the University of the Witwatersrand and requests for data can be made through <https://www.wits.ac.za/coe-human/open-access-datasets/>.

**Comment 7**. Throughout: Please include line numbers running throughout the text with the revised version.

**Response 7.** Line numbers have been added

**Comment 8**. Abstract: Please combine the Methods and Findings sections into one section, “Methods and findings”.
**Response 8.** These sections have been combined.

**Comment 9**. Abstract: Methods: We suggest emphasizing early on that the primary outcome of interest was suicidal ideation reported at any age.

**Response 9**. This has been specified, as suggested. The sentence now reads as follows (page 2, lines 36-37):

Suicidal ideation was self-reported at ages 14, 17, 22, and 28 years, and the primary outcome of interest was suicidal ideation reported at any age.

**Comment 10**. Abstract: Methods and Findings: Please quantify the main results (with 95% CIs and p values). Please mention the important dependent variables that are adjusted for in the analyses.

**Response 10.** We have added p-values alongside all confidence intervals for all reported Odds Ratios

**Comment 11**. Abstract: Methods and Findings: Please clarify “high birth order” briefly.

**Response 11.** We have clarified this as follows (page 2, lines 43-44):

higher birth order (i.e., increase in the order of birth in the family: first, second, third, fourth or later born child)

**Comment 12**. Abstract: Methods and Findings: In the last sentence of the Abstract Methods and Findings section, please describe the main limitation(s) of the study's methodology.
**Response 12.** We have added the following sentence to highlight the main limitations of the methodology (page 2, lines 50-52):

Main limitations of the study are the high attrition rate (61% of the original sample was included in this analysis) and the heterogeneity in the measurements of suicidal ideation.

**Comment 13**. Abstract: Conclusions: Please address the study implications without overreaching what can be concluded from the data; the phrase "In this study, we observed ..." may be useful.

**Response 13.** In line with this comment, we have modified our conclusion statement as follows (page 2, lines 53-55):

In this study from South Africa, we observed that early-life social and environmental adversities as well as childhood externalizing problems are associated with increased risk of suicidal ideation during adolescence and early adulthood.

**Comment 14**. Author summary: At this stage, we ask that you include a short, non-technical Author Summary of your research to make findings accessible to a wide audience that includes both scientists and non-scientists. The Author Summary should immediately follow the Abstract in your revised manuscript. This text is subject to editorial change and should be distinct from the scientific abstract. Please see our author guidelines for more information: <https://journals.plos.org/plosmedicine/s/revising-your-manuscript#loc-author-summary>
**Response 14.** We have added the author summary in our revised manuscript (page 3, lines 61-85)

**Comment 15**. Throughout: Please use square brackets for in-text citations, rather than superscript numbers. Please place the reference before the sentence punctuation, and please do not include spaces within brackets where multiple references are indicated, for example [1,2].
**Response 15.** This has been modified

**Comment 16**. Methods: Please ensure that the study is reported according to the STROBE guideline, and include the completed STROBE checklist as Supporting Information. Please add the following statement, or similar, to the Methods: "This study is reported as per the Strengthening the Reporting of Observational Studies in Epidemiology (STROBE) guideline (S1 Checklist)."

The STROBE guideline can be found here: <http://www.equator-network.org/reporting-guidelines/strobe/>

When completing the checklist, please use section and paragraph numbers, rather than page numbers.
**Response 16.** We have added the suggested sentence at the end of the methods section (page 10, lines 249-250) and provided the completed STROBE checklist in supplementary material.

**Comment 17**. Methods: Did your study have a prospective protocol or analysis plan? Please state this (either way) early in the Methods section.

a) If a prospective analysis plan (from your funding proposal, IRB or other ethics committee submission, study protocol, or other planning document written before analyzing the data) was used in designing the study, please include the relevant prospectively written document with your revised manuscript as a Supporting Information file to be published alongside your study, and cite it in the Methods section. A legend for this file should be included at the end of your manuscript.

b) If no such document exists, please make sure that the Methods section transparently describes when analyses were planned, and when/why any data-driven changes to analyses took place.

c) In either case, changes in the analysis-- including those made in response to peer review comments-- should be identified as such in the Methods section of the paper, with rationale.

**Response 17**. The analysis protocol was decided during study group meetings, but there is no formal document describing the analysis plan. As recommended, we have indicated any change in the analyses following the peer review comments. We have also provided the results obtained in the multivariable analyses as initially performed in the supplementary material (Table S3). The new sentences read as follows:

Page 8, lines 192-194:

birth weight (considered as a continuous variable measured in kg following a peer-reviewer’s remark; previously dichotomized into low, <2.5kg, vs non-low, ≥2.5 kg)

Page 10, lines 241-244:

This approach to the multivariable modelling was implemented following the comment of a reviewer. Initially, only variables that showed evidence of an association (*P*<0.05) in the univariable analysis were used in the multivariable model. The two methods led to consistent results.

Page 12, lines 293-294:

Multivariable analysis including only factors associated with suicidal ideation at *P* < 0.05 in the univariable analyses yielded consistent results (Table S3).

**Table S3.** Multivariable associations between childhood factors and suicidal ideation using factors associated with suicidal ideation in the univariable analysis at *P* < 0.05

|  | OR (CI) | *P* |
| --- | --- | --- |
| **Males** |  |  |
| Socioeconomic adversity score | **1.13 (1.02-1.25)** | **0.026** |
| Birth order (trend) | 1.02 (0.93-1.12) | 0.678 |
| Externalizing problems | **1.22 (1.08-1.39)** | **0.001** |
| Internalizing problems | 1.11 (0.98-1.26) | 0.093 |
|  |  |  |
| **Females** |  |  |
| Low birth weight | **1.33 (1.05-1.68)** | **0.044** |
| ACE score | **1.11 (1.01-1.21)** | **0.023** |
| Birth order (trend) | **1.14 (1.06-1.23)** | **< 0.001** |
| Externalizing problems | **1.16 (1.03-1.31)** | **0.010** |

**Comment 18**. Methods: Please provide some brief details on how mothers were recruited for the Bt20+ cohort.

**Response 18.** As suggested, we have provided the following sentence to detail the way in which mothers were recruited and referenced the cohort profile of the study, which gives further details on the recruitment process.

The added sentence reads as follows (page 6, lines 145-147):

Mothers were recruited from public antenatal clinics in the area in late 1989, when researchers began interviewing women who were predicted to deliver their babies during the study’s enrolment period.

**Comment 19**. Methods: Please clarify the information on phrasing of questions across languages: “Data
collection was performed mainly in isiZulu, Sesotho, or English, with consensual agreement on the
phrasing of questions asked across the different languages.”

**Response 19.** We have clarified the phrasing of this sentence, which now reads as follows (page 6, lines 152-155):

To overcome potential language problems, data collection was performed in the participant’ language of choice (isiZulu, Sesotho, or English); consensual agreement on the phrasing of questions in the different languages was reached for each item when instrument validation was performed [34].

**Comment 20**. Methods: “For this study, we analyzed data from a sample of 2,020 participants with at least one measure of suicidal ideation at ages 14, 17, 22, or 28 years. This analytical sample differed from the original cohort on a number of variables, including maternal age and schooling, household crowding, and assets. Inverse probability weighting was therefore used in all analyses to address biases due to differential attrition.”
Please provide a table comparing the sample of 2,020 participants included here and the original cohort. A participant flowchart would be helpful.

**Response 20.** In response to this suggestion, we have provided a table comparing included and non-included participants in the supplementary material (Table S1) and referred to it in the text (page 6, line 158). The table is reported below:

**Table S1.** Comparison of the characteristics of participants included and nonincluded in the analysis sample

|  | Included participants  (N=2020) | Nonincluded participants  (N=1253) | *P* |
| --- | --- | --- | --- |
| Birth weight (Kg), mean (SD) | 3.06 (0.51) | 3.08 (0.52) | 0.166 |
| Child male sex, n (%) | 1042 (51.6) | 637 (50.8) | 0.705 |
| Low maternal age, n (%) | 149 (7.4) | 52 (4.2) | <0.001 |
| Low maternal education, n (%) | 1096 (59.1) | 615 (57.0) | 0.271 |
| Household crowding, n (%) | 772 (44.6) | 249 (27.1) | <0.001 |
| Poverty, n (%) | 890 (44.1) | 598 (47.7) | 0.044 |
| Material deprivation, mean (SD) | 0.15 (0.92) | -0.62 (1.07) | <0.001 |
| Loss, mean (SD) | 0.07 (0.99) | -0.30 (0.99) | <0.001 |
| Family dynamics, mean (SD) | 0.12 (1.00) | -0.49 (0.81) | <0.001 |
| Abuse and violence, mean (SD) | 0.00 (1.01) | 0.04 (0.91) | 0.762 |
| Postnatal maternal depression, mean (SD) | 199 (17.2) | 127 (17.8) | 0.785 |
| Birth order, mean (SD) | 2.11 (1.08) | 2.15 (1.06) | 0.283 |
| Parity, mean (SD) | 2.18 (1.23) | 2.22 (1.20) | 0.385 |
| Previous abortions/stillbirths, n (%) | 219 (10.8) | 166 (13.2) | 0.043 |
| Externalizing problems, mean (SD) | 0.01 (0.98) | -0.07 (1.07) | 0.166 |
| Internalizing problems, mean (SD) | 0.08 (0.97) | -0.41 (1.07) | <0.001 |

The criteria for including participants were to have any data on suicidal ideation, therefore the flowchart is as follows:

We would be happy to include this figure in the supplementary material if the editor judges it helpful. Please, advice.

**Comment 21**. Ethical approval: Please clarify if this report is a secondary analysis of data collected from the Bt20+ study and if ethical approval for this analysis was waived.

Please clarify the nature of written consent from all participants at all waves- was written consent obtained from parents/guardians with written assent from the children, with written consent provide at waves conducted during adulthood?

Please also note whether and how individuals indicating having experienced suicidal ideation were followed up for additional intervention and care.

**Response 21.** The following three sentences were added to the methods section in response to this comment:

Pages 6-7, lines 162-169:

Ethical approval for the Bt20+ study was obtained from the Committee for Research on Human Subjects at the University of Witwatersrand, South Africa. Ethical clearance for the use of secondary data is only applicable where use of the data is not covered by primary data collection (the Bt20+ study) ethics approval. As this study is based on secondary data analysis within the parameters of the primary data collection’s ethics clearance, no further ethical approval is necessary.

All participants gave written informed consent at each individual data collection wave. Written consent was obtained by parents or guardians when children were minors, with verbal assent from children. From ages 16 to 28 Bt20+ participants provided written consent for each individual data collection wave.

Page7, lines 182-188

Follow-up intervention of care for participants was conducted at two points. Firstly, fieldworkers indicated if a participant appeared to be under psychological distress in study notes immediately after administration of the questionnaire, which were reviewed at the end of each day of data collection. Secondly, during data entry, a built-in calculation on items probing for ‘suicide ideation in the past month’ gave a positive for a ‘mental health referral needed’ variable. Participants expressing distress were accordingly put in contact with social and health services linked to the study.

**Comment 22**. Methods: Study waves at age 17 and 22: Please clarify if the response of “No more than usual” to “Have you recently found that the ideas of taking your own life kept coming into your mind?” was dichotomized as yes or no, and if categorized as “no” please explain as this seems as if it could indicate suicidal ideation.

**Response 22.** This comment made us realize that there was a mistake in our text, as the response options for the item used to evaluate suicidal ideation with the General Health Questionnaire are: “definitely not”, “I don’t think so”, “has crossed my mind”, and “definitely yes” (Goldberg and Hillier, *Psychological Medicine*, 1979, 9, 139-145) rather than the ones initially reported. We apologize for this mistake. “Definitely not” and “I don’t think so” were categorized as “no suicidal ideation”, while “has crossed my mind” and “definitely yes” were considered indicative of suicidal ideation. This has been amended in the text (page 7, lines 176-179).

Thank you for noticing this.

**Comment 23**. Methods: It would be helpful to include copies of the relevant questions used to obtain the primary outcome and childhood risk factor data, as supporting information.
**Response 23.** The questions assessing suicide ideation at ages 14, 17, 22 and 28 are included in the methods section of the manuscript. We have also collated each of the items and their response options in the supplementary material (Supplemental Appendix 1). The following sentence has been added to the methods section to refer to the new supplementary appendix (page 8, lines 190-191):

The following childhood risk factors were investigated (see also Supplementary Appendix 1 for the items used to assess these variables):

**Comment 24**. Methods: Please describe the inverse probability weighting that was used to account for study attrition. Please include more description of the multiple imputation methods. It would be helpful to indicate the amount of missing responses for each relevant outcome/factor within a Table.
**Response 24.** As suggested, we added more description of the inverse probability weighting and the multiple imputation methods. The following has been added in the revised manuscript.

Page 6, lines 160-162:

Weights were derived from the independent baseline characteristics (maternal age, maternal schooling, household crowding, assets) predicting inclusion in the sample using a logistic regression model’s individual predicted probabilities [35].

Page 10, lines 244-248.

To account for missing data in childhood factors, we estimated our model using multiple imputations: 50 imputed datasets were generated using the Amelia II package in R, which relies on a bootstrap expectation-maximization algorithm to impute missing multivariate data [46]. Models were then estimated across all imputed datasets and results pooled. The amount of missing data is shown in **Table S2**.

|  | n (%) |
| --- | --- |
| Birth weight (Kg), mean (SD) | 4 (0.2) |
| Child male sex, n (%) | 0 |
| Low maternal age, n (%) | 2 (0.1) |
| Low maternal education, n (%) | 167 (8.3) |
| Household crowding, n (%) | 290 (14.4) |
| Poverty, n (%) | 0 |
| Material deprivation, mean (SD) | 138 (6.83) |
| Loss, mean (SD) | 140 (6.93) |
| Family dynamics, mean (SD) | 158 (7.82) |
| Abuse and violence, mean (SD) | 897 (44.4) |
| Postnatal maternal depression, mean (SD) | 865 (42.8) |
| Birth order, mean (SD) | 0 |
| Parity, mean (SD) | 0 |
| Previous abortions/stillbirths, n (%) | 0 |
| Externalizing problems, mean (SD) | 399 (19.7) |
| Internalizing problems, mean (SD) | 398 (19.7) |

**Comment 25**. Methods: Please specify the significance level used (e.g., P<0.05, two-sided).

**Response 25.** The following has been added (page 10, lines 248-249):

Analyses were performed in R version 3.6, and the statistical significance level used was P<0.05, two sided.

**Comment 26**. Results: Please provide an analysis comparing characteristics of those for which data were missing compared with those included in the study.

**Response 26.** We provided this analysis in Table S1, now referred to in the methods section (page 6). See also our Response to the Editor’s Comment 20.

**Comment 27**. Results: For all results presented in the text (e.g from univariable and multivariable analyses) please present both the 95% CIs and p values. Please present the overall associations for each factor. If results stratified by sex are presented, please present the findings for both sexes, and please also report the interaction.
**Response 27.** As suggested, we have reported all P values for both the univariate and univariate analyses, results for both sexes, as well as the P value for the interaction in the revised manuscript (see pages 10 and 11, whole results section).

**Comment 28**. Results: “This was especially true for poverty (1.25, CI 1.02- 1.52), low maternal education (OR 1.31, CI 1.04-1.64), and household crowding (OR 1.24, CI 0.98-1.57).” Please clarify that this describes the associations for males.
**Response 28.** This was clarified as follows (page 11, lines 271-272):

This was especially the case for poverty (OR for males: 1.25, CI 1.02-1.52, *P*=0.031; OR for females: 0.89, CI 0.76-1.04, *P*=0.152; *P_interaction_* = 0.010)~~, low maternal education (OR 1.31, CI 1.04-1.64), and household crowding (OR 1.24, CI 0.98-1.57)~~

Please, note that we have not reported the estimates for low maternal education and household crowding in this sentence to avoid overemphasizing sex differences not supported by a statistically significant interaction, as suggested by the reviewers and editor.

**Comment 29**. Results: Childhood internalizing problems: For differences reported between males and females, it seems as if the interaction by sex did not reach statistical significance. Please mention in the text that while the association with internalizing problems was significant for males (and please report the result for females) there was no evidence to support an interaction effect by sex. Please present the overall associations for externalizing and internalizing problems in addition to the sex-stratified analyses.

**Response 29.** This has been amended as suggested. The sentence now reads as follows (page 11, lines 273-281):

Both externalizing (OR 1.15, CI 1.06-1.25, *P*<0.001) and internalizing (OR 1.12, CI 1.04-1.21, *P=*0.003) problems in childhood were associated with increasing odds of suicidal ideation. In subgroup analyses, childhood externalizing problems were associated with an increased likelihood of reporting suicidal ideation among both males (OR 1.28, CI 1.13-1.45, *P*<0.001 for 1-SD increase in externalizing problems score) and females (OR1.19, CI 1.06-1.34, *P*=0.001; *P_interaction_* = 0.380), while internalizing problems were associated with suicidal ideation among males only (OR for males: 1.19, CI 1.05-1.34; *P*=0.003; OR for females: 1.05, CI 0.95-1.16, *P*=0.328), although the interaction did not reach statistical significance (*P_interaction_* = 0.118).

**Comment 30**. Discussion: Please present and organize the Discussion as follows: a short, clear summary of the article's findings; what the study adds to existing research and where and why the results may differ from previous research; strengths and limitations of the study; implications and next steps for research, clinical practice, and/or public policy; one-paragraph conclusion.
**Response 30.** In the revised manuscript, we have organized the Discussion section as suggested and used sub-headings to facilitate the organization of the text (pages 12-19).

**Comment 31**. Discussion: Please temper statements related to primacy with “To the best of our knowledge” or similar: “This is the first study to prospectively examine associations…”

**Response 31.** As suggested, we rephrased the sentence as follows (page 12, line 317):

To the best of our knowledge, this is the first study to prospectively examine…

**Comment 32**. Discussion: “A key strength of this paper is the use of longitudinal data from the longest birth cohort in Africa to prospectively examine the impact of the early childhood environment on suicidal ideation in youth.” Here and throughout, please revise to avoid language that implies causality. Instead, please refer to associations.

**Response 32.** We have revised the text and corrected this and other instances in which our language implied causality and now refer to *associations* instead.

**Comment 33**. Figure 1: Please present this information in a table format. Please note the numbers as well as percentages reported for both males and females. Please indicate in the legend what is represented by the “error bars” and please note that the percentages are relative to the individuals in the cohort for which suicidal ideation data were available at the given age.
**Response 33.** As suggested, we provided the information in a table format as showed below. We renumbered the tables to maintain the order of appearance in the text.

**Table 2.** Prevalence rates of suicidal ideation in the Birth to Twenty Plus cohort

|  | Entire sample | |  | By sex |  |  |  |  |  |
| --- | --- | --- | --- | --- | --- | --- | --- | --- | --- |
|  |  | |  | **Females** |  | **Males** |  | **Sex comparison** |  |
| Age | n/N | % (95% CI) |  | n/N | % (95% CI) | n/N | % (95% CI) | RR (95% CI) | *P* |
| 14 | 94/1209 | 7.8 (6.3-9.3) |  | 71/632 | 11.2 (8.8-13.7) | 23/577 | 4.0 (2.4-5.6) | 2.81 (1.79-4.61) | < 0.001 |
| 17 | 231/1532 | 15.1 (13.3-16.9) |  | 161/814 | 19.8 (17-22.5) | 70/718 | 9.8 (7.6-11.9) | 2.03 (1.54-2.7) | < 0.001 |
| 22 | 164/1582 | 10.4 (8.9-11.9) |  | 105/821 | 12.8 (10.5-15.1) | 59/761 | 7.8 (5.9-9.6) | 1.65 (1.20-2.28) | 0.001 |
| 28 | 96/1388 | 6.9 (5.6-8.3) |  | 58/728 | 8.0 (6.0-9.9) | 38/660 | 5.8 (4.0-7.5) | 1.38 (0.92-2.10) | 0.130 |

The table shows the number of participants reporting suicide attempt (n) relative to the number of assessed participants (N) at any assessment age, as well as the rate (%) with 95% confidence interval (CI). Statistics are provided for the entire sample and for females and males separately. The prevalence of suicidal ideation in females vs. males is compared using Risk Ratio with 95% confidence interval, and *P* values were computed using chi-square tests. All tests are two-sided and considered statistically significant at *P* < 0.05.

Additionally, we provided the following legend to Figure 1:

The figure illustrates the prevalence of suicidal ideation at each assessment occasion and their change over time. Percentages are relative to the individuals in the cohort for which suicidal ideation data were available at the given age, namely: 1209 (632 females, 577 males), 1532 (814 females, 718 males), 1582 (821 females, 761 males), and 1388 (728 females, 660 males) participants at ages 14, 17, 22, and 28 years respectively. Vertical bars represent 95% confidence intervals around the point prevalence.

**Comment 34**. Figure 2: Please provide a descriptive legend describing the figure, including how risk ratio was determined and the meaning of the points and bars.
**Response 34.** We have provided the following legend:

The figure represents the comparisons of suicidal ideation rates in females vs males as Risk Ratios (RR) at each age. RR were calculated by dividing the prevalence rate in females by the prevalence rate in males, e.g., for age 14 years: (71/632) / (23/577) = 2.81. Vertical bars represent 95% confidence intervals of the RR. The horizontal dashed line represents the null (RR=1), and confidence intervals bars indicates that the RR is not statistically significant at *P* < 0.05, except at age 28 years.

**Comment 35**. Table 1: Please specify the significance level used (eg, P<0.05, two-sided) and the statistical test used to derive the p value.
**Response 35.** The following has been added:

All tests are two-sided and considered statistically significant at P < 0.05. P values have been obtained using Student t-tests and chi-square tests for continuous and categorical variables, respectively.

**Comment 36**. Table 2: Please provide the p values for the associations for the whole sample, males, and females. In the legend, please note the statistical tests used.
**Response 36.** We have added all P values, as suggested, and the statistical test used was specified. The legend now reads as follows (now renumbered as Table 3):

All tests are two-sided and considered statistically significant at P < 0.05. P values have been obtained using univariable logistic regressions.

**Comment 37**. Table 3: Please provide the p values for these associations. In the legend, please note the statistical tests used.

**Response 37.** We have added all P values, as suggested, and the statistical test used was specified. The legend now reads as follows (now renumbered as Table 4):

All tests are two-sided and considered statistically significant at P < 0.05. P values have been obtained using multivariable logistic regressions.

**Comment 38**. References: Please use the "Vancouver" style for reference formatting, and see our website for other reference guidelines <https://journals.plos.org/plosmedicine/s/submission-guidelines#loc-references>
**Response 37.** This has been amended. In the revised version, the references style “Vancouver” is used.

**Reviewer #1**

**Comment 1.** I confine my remarks to statistical aspects of this paper.  The general approach is fine, but I have a few issues to resolve before I can recommend publication.
**Response 1.** Thank Dr. Flom for providing your feedback on the statistical aspect of our paper, and for suggesting useful ways to improve our analysis. Please, find below our responses to your comments.

**Comment 2.** Abstract (and similar in main text) - when comparing males to females be careful to not accept the null. You can either insert a bunch of "significantly" or else, rather than say "not associated" say things like "higher in males" or "higher in females".
**Response 2.** Thank you for this important comment, to which we agree. We have modified our abstract and main text as suggested. For example, the abstract now reads as follows (page 2, lines 45-50, ***in document with tracked changes***):

Socioeconomic adversity (OR 1.13, CI 1.01-1.27, *P*=0.031) was significantly associated with suicidal ideation among males only, while birth weight (OR 1.20, CI 1.02-1.41, *P*<0.030), ACEs (OR 1.11, CI 1.01-1.21, *P*=0.030), and higher birth order (OR 1.15, CI 1.07-1.243, *P*<0.001) were significantly associated with suicidal ideation among females only. Externalizing problems in childhood were significantly associated with suicidal ideation among both males (OR 1.23, 1.08-1.40, *P*=0.002) and females (OR 1.16, CI 1.03-1.30, *P*=0.011).

Please note that in the abstract we reported the significant associations in males and females separately, avoiding direct comparisons. We have not reported interactions and nonsignificant ORs in the abstract to avoid making the text difficult to read. However, in the Result section of the main text, we reported all interactions and non-significant ORs for the other sex, and rephrased the language as recommended (see pages 10-11).

**Comment 3.** p. 3   I'm not sure you can say that preventing suicidal ideation would enhance mental well being. It could be the other way around. 
**Response 3.** In response to this comment, we modified our sentence as follows (page 4, lines 92-93):

These data suggest that preventing suicidal ideation is critical to reduce suicide risk.

**Comment 4.** "seriously considering and attempting suicide" - which one?  Either?
**Response 4.** We have clarified the sentence, which now reads as follows (page 4, lines 96-97):

with 1 in 5 adolescents reporting seriously considering ~~and attempting~~ suicide in the past 12 months.

**Comment 5.** p. 6  Don't categorize birth weight and (maybe) don't categorize depression.  Categorizing a continuous variable is nearly always a bad idea. See my blog post <https://medium.com/@peterflom/what-happens-when-we-categorize-an-independent-variable-in-regression-77d4c5862b6c> 
**Response 5.** Thank you for this comment. We now reported our estimates for birth weight as a continuous variable (depression is already considered as such). Our conclusions remained unchanged (see Table 3 and Table 4 as well as the result section).

**Comment 6.** p. 7  This method of model building is known as bivariate screening and it is not recommended. All the results will be wrong. P values are too low, standard errors are too small, parameter estimates are biased away from 0.  See Harrell, Regression Modeling Strategies.
**Response 6.** We agree with this comment and thank Dr. From for having pointed it out. We have changed the way in which we built our model. Instead of using bivariate screening, we now considered all variables in our multivariable model, excluding collinear variables (e.g., birth order and parity). Results are overall unchanged (see Table 4), i.e., estimates are very similar to the previous model. For transparency, we reported our previous model in Supplementary Table S3.

The data analysis section of the revised version of the paper now reads as follows (pages 9-10, lines 237-241):

We systematically tested the interaction between each factor and child sex and report analyses separately for males and females. Then, variables were jointly entered in a multivariable logistic regression model to estimate their independent associations with suicidal ideation, avoiding the use of collinear variables.

Peter Flom 

**Reviewer #2**

This is a well-written manuscript focusing on an important subject in child mental health. The research aims to show the possible impact of individual, familial and environmental factors on the suicidal ideation of young people in South Africa taken from a cohort sample. 

**Comment 1.** * Are the claims properly placed in the context of the previous literature? Have the authors treated the literature fairly? 
Discussion was good and comprehensive. 
**Response 1.** We thank the reviewer for providing feedback to our paper and for the positive comments.

* Do the data and analyses fully support the claims? If not, what other evidence is required? 
**Comment 2.** Overall, the sample and methods of the study are robust. However, could the authors explain why they have not used ACE-IQ to find out about adverse childhood experiences?

**Response 2.** We thank the reviewer for these comments on the ACE measure. In the Birth to Twenty Plus cohort, ACEs were prospectively measured during early and middle childhood before the development of the ACE-IQ. The ACE-IQ was later developed as a retrospective measure of ACEs to be completed by adults and is based on the CDC-Kaiser ACEs scale. The 43 items of the ACE-IQ scale include all the items from the ACEs scale used in this study, with additional adversities, and the remaining items probe socio-demographics, family environment, relationships with parents and significant other, all of which are included in the Birth to Twenty Plus main questionnaire administered during routine data collection.

**Comment 3.** Furthermore, why did they not ask for ACEs after age 13? I believe this might have changed the cumulative ACE score and therefore its possible association with suicidal ideation.

**Response 3.** Our primary interest in this paper was to investigate the association between childhood factors and adolescence and young adulthood suicidal ideation. Therefore, although a measure of ACEs is available at age 28 in the Birth to Twenty Plus cohort, this measure was not used in this study. Additionally, our outcome was suicidal ideation at age 14-28, therefore all our predictors were measured before age 14 in order to respect the temporal precedence of predictors over outcomes.

**Comment 4.** What is the mean ACE score for the sample? Why did the authors use cumulative ACE score rather than the mean score which is more commonly used in the field? Have the authors looked into the impacts of individual ACE components on suicidal ideation?
**Response 4.** Thank you for this very pertinent comment. Indeed, ACEs are measured in different ways in the literature. In this study, we followed the cumulative risk model, which is one of the dominant models of the effects of developmental adversity on later health. This model states that it is the accumulation of adverse events, rather than the exposure to specific events, that is detrimental to health (Evans et al. 2013 Psych Bull). This model is commonly implemented in the ACEs literature using cumulative numeric score (the ‘ACE Score’) that indicates the total number of exposures, which is the approach that we followed.

In response to the reviewer’s comment, we added the following sentence to the methods section (page 9, lines 231-233, ***in document with tracked changes***):

This follows the cumulative risk model of ACEs, which states that it is the accumulation of adverse events, rather than the exposure to specific events, that is detrimental to health.

The mean of the standardized ACE score is reported in Table 1; we have now added the unstandardized one (see Table 1). The association between the ACE components and suicidal ideation is reported in Table 3.

**Comment 5.** The authors also mention about attrition being substantial despite being comparable with other cohorts. Have the authors compared baseline characteristics of participants who were lost to follow-up with clinical characteristics of those remaining? If so, what are the results, and could they have an effect on the results of the paper?
**Response 5.** This is an important comment, and we agree that this is an important piece of information to add. Therefore, the following table has been added to the manuscript as supplementary material (Table S1).

We refer to this table in the methods section alongside a brief description of the main baseline variables differentiating missing and non-missing participants, as follows:

This analytical sample differed from the original cohort on a number of variables, including maternal age and schooling, household crowding, and assets (**Table S1**).

|  | Included participants  (N=2020) | Nonincluded participants  (N=1253) | *P* |
| --- | --- | --- | --- |
| Birth weight (Kg), mean (SD) | 3.06 (0.51) | 3.08 (0.52) | 0.166 |
| Child male sex, n (%) | 1042 (51.6) | 637 (50.8) | 0.705 |
| Low maternal age, n (%) | 149 (7.4) | 52 (4.2) | <0.001 |
| Low maternal education, n (%) | 1096 (59.1) | 615 (57.0) | 0.271 |
| Household crowding, n (%) | 772 (44.6) | 249 (27.1) | <0.001 |
| Poverty, n (%) | 890 (44.1) | 598 (47.7) | 0.044 |
| Material deprivation, mean (SD) | 0.15 (0.92) | -0.62 (1.07) | <0.001 |
| Loss, mean (SD) | 0.07 (0.99) | -0.30 (0.99) | <0.001 |
| Family dynamics, mean (SD) | 0.12 (1.00) | -0.49 (0.81) | <0.001 |
| Abuse and violence, mean (SD) | 0.00 (1.01) | 0.04 (0.91) | 0.762 |
| Postnatal maternal depression, mean (SD) | 199 (17.2) | 127 (17.8) | 0.785 |
| Birth order, mean (SD) | 2.11 (1.08) | 2.15 (1.06) | 0.283 |
| Parity, mean (SD) | 2.18 (1.23) | 2.22 (1.20) | 0.385 |
| Previous abortions/stillbirths, n (%) | 219 (10.8) | 166 (13.2) | 0.043 |
| Externalizing problems, mean (SD) | 0.01 (0.98) | -0.07 (1.07) | 0.166 |
| Internalizing problems, mean (SD) | 0.08 (0.97) | -0.41 (1.07) | <0.001 |

It is difficult to know in which way attrition may have affected our results. However, attrition has certainly limited the possibility to generalize our results to the initial population, despite our effort to reduce bias due to attrition using inverse probability weighting. Therefore, as noted in our strengths and limitations section, caution should be used in generalizing the results to the South African population.

**Comment 6.** *	PLOS Medicine encourages authors to publish detailed methods as supporting information online. Do any particular methods used in the manuscript warrant such publication? If a protocol is already provided, for example for a randomized controlled trial, are there any important deviations from it? If so, have the authors explained adequately why the deviations occurred?
I have not seen any information about this. 
**Response 6.** The analysis protocol for this study was not published.

**Comment 7.** * Is this paper outstanding in its discipline? If yes, what makes it outstanding? If not, why not?
It is an important paper taken from a cohort sample, looking into an important subject in mental health, and aiming to find out the factors associated with suicidal ideation in a population from a low/middle income country.
**Response 7.** We thank the reviewer for highlighting the importance of our paper.

**Comment 8.** * Does the study conform to any relevant guidelines such as CONSORT, MIAME, QUORUM, STROBE, and the Fort Lauderdale agreement?
N/A
**Response 8.** According to the Editorial Office Comment #16, we have now provided the STROBE checklist in the supplementary material.

**Comment 9.** * Are details of the methodology sufficient to allow the experiments? Yes. 
**Response 9.** Thank you. We further improved the methodological details following the comments that we received during this round of review.

**Comment 10.** * Is any software created by the authors freely available? 
The dataset is not available online. Authors state that the data underlying the results presented in the study are available to authorized researchers and provide a link to apply for authorization to access data.
**Response 10.** We clarified the data access policy as requested by the Editor (see Response #6 to the Editor’s comment).

**Comment 11.** * Is the manuscript well organized and written clearly enough to be accessible to non-specialists? 
The manuscript is well-written and easy to understand. The English and Scientific language is of adequate quality throughout the manuscript. 
**Response 11.** Thank you for this and the other comments provided.

**Reviewer #3**

We thank the reviewer for the positive comments on our paper.

Introduction.
**Comment 1.** Please elaborate or define or give examples of internalizing and externalizing problems for the benefit of the reader. Especially since externalizing factor is one of the significant findings in this study.
**Response 1.** As suggested, we added a definition of externalizing and internalizing problems. The text now reads as follows (page 4, lines 102-105, ***in document with tracked changes***)

Beyond the contribution of childhood externalizing problems (i.e., behaviors such as conduct disorder and hyperactivity/impulsivity which are overt and can result in conflict with others)[9] and internalizing problems (i.e., emotional symptoms such as anxiety or depression which reflect internal distress) [9-15]

Added Reference: Cicchetti D, Toth SL. *Internalizing and Externalizing Expressions of Dysfunction: Volume 2*. Psychology Press; 2014.

Methods
**Comment 2.** It is quite a complicated methodology with various timelines, from participant and mothers as well as various questionnaires used. Perhaps a diagrammatic flowchart will benefit the readers in understanding the process.
**Response 2.** Thank you, this is a useful suggestion. We have provided the following chart to clarify the timeline of our exposure and outcome variables as Supplemental Figure S1:

**Comment 3.** A few of the childhood risk factors were elicited at time of child's birth such as birth order and socioeconomic adversity, which may have changed during their lifetime. This step cannot be undone but can be mentioned as a limitation of this study.
**Response 3.** We agree with this comment and have added the following sentence to the limitation section (page 17, lines 434-435):

Another limitation is that some of the childhood factors were only measured once, and our analysis did not take into account their time-varying nature (e.g., socioeconomic adversity).

Childhood internalizing and externalizing problems: 
**Comment 4.** It is not very clear regarding SACAS. You mentioned in the manuscript:" The South African Child Assessment Schedule (SACAS) was used to ascertain child externalizing problems……The SACAS is an 85-item questionnaire based on the Child Behaviour Checklist" and "Externalizing problems were assessed with 35 items describing…"
Is the 35 items part of SACAS? 
**Response 4.** We agree that this section was unclear. Externalizing and internalizing scores were computed from the SACAS items. We have clarified the paragraph, that now reads as follows (pages 8-9, lines 213-218):

The SACAS is a questionnaire based on the Child Behavior Checklist [43], and was adapted idiomatically to the South African culture and translated into isiZulu, Sotho, and Afrikaans [40]. Externalizing problems were assessed with 35 items from the SACAS describing aggressive and rule-breaking behaviors (e.g., “Is he/she disobedient at school?”, “Does he/she physically attack people?”). Internalizing problems were assessed with 32 items from the SACAS describing anxiety and depressive symptoms (e.g., “Is he/she sad or depressed?”, “Is he/she too fearful or anxious?”).

**Comment 5.** Perhaps can put in the internal reliability (Cronbach alpha) of the SACAS to further strengthen the strength of this tool
**Response 5.** Cronbach alpha for internalizing and externalizing scores have been added as follows (page 9, lines 221-223):

In our sample, both externalizing and internalizing problems measures showed good reliability (Cronbach’s alpha were 0.80-0.85 and 0.76-0.80, respectively).

**Comment 6.** Throughout the study, there seems to be different ages at which the assessments took place which does not tally with each other. For example:
- Child externalizing problems were assessed at 5,7, 10 from their mothers.
- Adverse Childhood Experiences (ACEs) were assessed at 5,7, 11
Why is this so?
**Response 6.**  The current Birth to Thirty Plus study began as Birth to Ten (Bt10) and was funded for a further 10 years in the Birth to Twenty (Bt20) phase. Bt10 broadly surveyed children's health and development. Bt20, while continuing to collect a wide range of data related to young people's health and development, was targeted towards answering specific questions related to risk associated with lifestyle, cardiovascular disease, and diabetes. Year 10 data collection marked the end of the first phase, and Year 11 the beginning of the second phase. Questions on ACEs were not included in the year 10 wave but were included in the Year 11 wave given the focus on risk factors in this second phase.

Adverse Childhood Experience (ACEs)
**Comment 7.** Are the questions to assess ACEs from a reliable and validated questionnaire?
**Response 7.** The ACEs scale was part of a larger “life events” questionnaire which probed a broad range of negative events. Individual items that formed the ACEs scale were pulled to complete the ACEs dataset. While there is some debate on the range of adversities that should be measured, the ACE scale is a measure of exposure to traumatic stress in childhood based on a set of common exposures and is considered epidemiologically credible and feasible for estimating prevalence and public health burden (Anda RF, et al. *American Journal of Preventive Medicine*. 2010). The items of this scale were later included in scales developed to assess ACEs, such as the ACE-IQ scale (WHO).

**Comment 8.** "Overall exposure to ACEs was computed by summing the number of reported ACEs (by either mothers or participants) and the final score was standardized" …is not a very clear description of the scoring process.

**Response 8.** This has been clarified as follows (page 9, lines 229-233):

The overall level of exposure to ACEs was computed by summing the number of reported ACEs (by either mothers or participants). This follows the cumulative risk model of ACEs, which states that it is the accumulation of adverse events, rather than the exposure to specific events, that is detrimental to health. The final score was then standardized (z-score transformed).

**Reviewer #4**

**Comment 1.** This is a well-written paper that investigates an important public health problem about which little is known, re: childhood risk factors for youth suicide (14-28 years) in a LMIC setting (South Africa). The study data is from a population-based longitudinal prospective study that followed-up children from birth to adulthood. Study methods, measures and data analyses are adequately described. Suicidal ideation (outcome measure) was assessed as self-reported suicidal ideation at any time (at ages 14, 17, 22, and 28 years). Independent measures included early-life adversity (data collected at baseline), children's externalizing and internalizing problems (assessed at ages 5, 7, and 10 years, from maternal reports using the SACAS), and ACEs (obtained from mothers at child ages 5, 7, and 11, and from children at ages 11 and 13). The main findings were:
1.	22.3% participants reported SI between ages of 14 and 28; peak SI was at age 17 years
2.	Females reported higher SI at all time points except for age 28 when males = females
3.	Externalizing problems in childhood independently predicted SI
4.	Internalizing problems in childhood was not an independent predictor of S
5.	Childhood socioeconomic adversity was associated with SI in males alone 
6.	ACEs, low birth weight, and high birth order, were associated with SI in females alone
**Response 1.** We thank the reviewer for these positive comments on our paper.

**Comment 2.** The lack of an association between internalizing behaviors and risk of suicide is a major albeit surprising finding that is inconsistent with existing literature (1). This discrepancy raises the question of the extent to which the study (measures, procedures) establishes a trustworthy cause and effect relationship between internalizing behaviors and suicidal ideation. That is, the study's internal validity that makes it possible to eliminate alternative explanations for the finding.

**Response 2.** This is an important comment, and we thank the reviewer for raising it. Although surprising, this finding is not inconsistent with the literature or the association between childhood behaviors and later suicidal risk.

Our study found an association for internalizing problems in the univariable analyses in the whole sample and in males only. However, for males, this association was no longer significant in the multivariable model, suggesting that the association for internalizing problems was *not independent* from the other variables in the model (although there was a trend towards statistical significance). The pattern was different from females, for which evidence clearly pointed to a lack of statistical association for internalizing problems in both univariable and multivariable analyses.

This pattern or results is consistent with the literature. For example, in two of our previous studies based on two independent Canadian samples (Orri et al. 2018 *JAMA Psych*; Commisso et al. 2021 *Psych Med*), we found that childhood internalizing behavior at 6-12 years alone are not independently associated with suicide risk, but only if in combination with externalizing problems. This is also consistent with other studies, such as one using a large sample form Finland (Sourander, *JAMA Psych* 2009) and another one using a large sample from the UK (Geoffroy et al., *Psych Med* 2014).

However, we do agree with the reviewer on the fact that some other studies suggested an independent association of internalizing problems with suicide risk, including the study form Auerbach and colleagues. Such studies however, usually focused on adolescence and adulthood, which is a period of the life course in which internalizing problems are more prominent than in early/middle childhood. Therefore, our pattern of results may be specific to internalizing problems measured early in life.

Finally, we think that it is worth noting that comparing our findings with previous studies is inherently difficult because of the lack of longitudinal studies in South Africa and other LMICs. The environment and cultural norms, including those related to the interpretation of some behaviors as being “frequent” or “problematic” for the participants in our cohort may significantly differ from those of participants in HICs, which calls for caution in comparing our results to those from prior studies.

In response to the reviewer’s comment, we added the following sentence to our discussion (page 14, lines 357-365 ***in document with tracked changes***):

This is consistent with previous studies in HICs that have shown that externalizing behavior and comorbid internalizing-externalizing behavior are more strongly associated with suicide-related outcomes than internalizing behavior alone [12,14,15]. While other studies found independent associations of internalizing behavior with suicide-related outcomes, these studies often measured internalizing behavior in adolescence rather than in childhood [53,54]. However, it is also important to consider that the lack of association for internalizing problems may be due to the developmental age in which internalizing problems have been measured, since some previous studies in HICs suggested associations between internalizing problems measured in adolescence and suicide-related outcomes.

Additionally, please, find below the point-by-point reply to the potential sources of bias raised by the reviewer:

Threats to internal validity and thus potential sources of bias include:

**Comment 3.** Use of a parent report measure to assess internalizing behaviors. Parent ratings of children's behaviors may reflect parental attitudes and stress just as much as they reflect the child's behavior

**Response 3.** We fully agree with this comment. Unfortunately, only mother reports were available in the Birth to Twenty Plus cohort during early/middle childhood. In response to the reviewer’s comment, we added the following limitation (page 17, lines 435-437):

Furthermore, only parent-reports of child behavior were available in early and middle childhood. This may have introduced measurement errors, especially for internalizing behavior measures that can be affected by maternal state of mind and attitudes.

**Comment 4.** Repeated testing of participants using the same measures. This influences study outcomes as participants will often do better as they become used to the testing process

**Response 4.** We agree with this comment. However, repeated testing is more likely to influence each other when the time between testing occasions is short (days or months). In our case, children’s problems were measured at age 5, 7, and 10, i.e., with a two-to-three-year interval. Additionally, repeated testing bias is often due to a learning effect and affects tests in which participants perform a task (e.g., neurocognitive testing), while in our case the participants’ behavior were rated by mothers and there was no task to learn. Therefore, it is unlikely that repeated testing significantly threats internal validity of our internalizing problems measure.

**Comment 5.** Maturation effects: this refers to the impact of time as a variable in a study (for example, participants growing older). This can make it impossible to rule out whether effects seen in the study were simply due to the effect of time.

**Response 5.** We agree that maturation effect may be at play when multiple measures of the same construct are performed over a period of rapid development, such as early and middle childhood. This is a known threat to internal validity for studies investigating the development of a behavior over time. However, in our case, we think it is unlikely that maturation effects constitute a bias for the association between internalizing problems and suicidal ideation, because we did not investigate changes in internalizing problems over time. Rather, we used the mean score of internalizing behavior between 5 and 10 years. This average measure is likely to capture the stability of internalizing symptoms over time, i.e., to rank children based on the average manifestation of internalizing problems between 5 and 10 years of age. Arguably, this approach may have increased internal validity because using multiple measures decreases the influence of fluctuations and measurement errors that usually affect single-occasion measurements.

**Comment 6.** Attrition: in the study, the analytical sample was a biased one as it differed from the original cohort on many variables, including maternal age and schooling, household crowding, and assets.

**Response 6.** We thank the reviewer for this comment. We agree that attrition may introduce important bias, especially regarding the generalizability of the findings to the population. As in most longitudinal cohort studies, non-random attrition is present in the Birth to Twenty Plus cohort. To reduce the influence of attrition bias, we used inverse-probability weighting, but we acknowledge that this is unlikely to fully address the potential bias. Therefore, caution should be used when interpreting the results. We have now added a table presenting the differences in baseline variables between included and nonincluded participants to be transparent on the characteristics of participants lost due to attrition (see Table S1).

**Comment 7.** Inverse probability weighting: used to address biases due to differential attrition in data analyses. However, one problem issue with IP-weighting is that participants who are extremely unlikely to be treated (that is, those with negative association for SI) will end up with a large weight, potentially making the weighted estimator highly unstable. A common alternative to the conventional weights that at least "kind of" addresses this problem are the stabilized weights, which use the marginal probability of treatment instead of 1 in the weight numerator (2).
**Response 7.** Thank you for this comment on IPW, with which we agree. As we did not observe any extremely unlikely weights, the use of stabilized or classical weights did not change our results (table below).

This suggests that the procedure used to calculate IPW is unlikely to explain the reported association between internalizing problems and suicidal ideation.

| **IPW** | **Males** | **Females** |
| --- | --- | --- |
| Regular | 1.12 (0.99-1.27) | 1.00 (0.90-1.11) |
| Stabilized | 1.12 (0.96-1.30) | 1.00 (0.88-1.13) |

I would recommend that the authors address these issues pertaining to internal validity.


References:
1.	Auerbach, R. P., Mortier, P., Bruffaerts, R., Alonso, J., Benjet, C., Cuijpers, P., Demyttenaere, K., Ebert, D. D., Green, J. G., Hasking, P., Lee, S., Lochner, C., McLafferty, M., Nock, M. K., Petukhova, M. V., Pinder-Amaker, S., Rosellini, A. J., Sampson, N. A., Vilagut, G., Zaslavsky, A. M., … WHO WMH-ICS Collaborators (2019). Mental disorder comorbidity and suicidal thoughts and behaviors in the World Health Organization World Mental Health Surveys International College Student initiative. International journal of methods in psychiatric research, 28(2), e1752. <https://doi.org/10.1002/mpr.1752>
2.	The intuition behind inverse probability weighting in causal inference <https://www.rebeccabarter.com/blog/2017-07-05-ip-weighting/>
