# Supplementary material for: Childhood factors associated with suicidal ideation among South African youth: A 28-year longitudinal study of the Birth to Twenty Plus cohort
Source: PLoS Med. 2022 Mar 15;19(3):e1003946. doi: 10.1371/journal.pmed.1003946 (PMC8923476; doi:10.1371/journal.pmed.1003946)
Supplement: Attachment — Submitted filename: 2022-02-09_R2_ResponseLetter.docx [file pmed.1003946.s008.docx]

**Requests from Editors:**

**Comment 1.** From the academic editor: The amount of missing data for violence/abuse and for postnatal depression was very high. So, although postnatal depression was not associated with attrition from the cohort, I wonder if it was associated with whether or not data were available on these variables. This may help to explain the lack of association between postnatal depression and later suicidality (although the authors' arguments about why that association may not be seen in this context are also relevant). Please address and comment on the potential relationship between the data that were not available and depression and how this could factor into the absence of a significant association with suicidal ideation.
**Response 1.** Thank you for this comment. We have added the following sentence in the discussion section to account for possible influence of missing data on these associations (lines 356-359 in the version with track changes):

However, maternal depression had a high rate of missing data in our sample (**Table S1**). Therefore, although maternal depression was not associated with attrition, caution should be used to interpret this lack of association, as the mothers with the highest depressive symptoms may have dropped out from the study.

However, although it is plausible that mothers with higher levels of depression did not answer the depression questionnaire (i.e., that the mothers for which depression score is unavailable would have scored higher in depression), this cannot be formally tested (and similarly for violence/abuse).

Perhaps we have misunderstood the academic editor’s suggestion. In this case, would it be possible to clarify the request? We would be happy to address it then.

**Comment 2**. Title: We suggest the following revision to the title. Please make this change in the manuscript submission system as well as the text: “Childhood factors associated with suicidal ideation among South African youth: A 28-year longitudinal study of the Birth to Twenty Plus cohort”
**Response 2.** We changed the title, as suggested.

**Comment 3**. Data availability statement: Please update this information in the manuscript submission system (Data availability section) rather than including it at page 20. Please note that there was no non-author contact information provided, as indicated in your response.
**Response 3.** This was updated. We removed the non-author contact upon request of the PLoS Medicine editorial team, which contacted us right after submitting the revised version of the manuscript. We however forgot to amend the response letter. Please do not hesitate to let us know if you prefer us to provide the email of a non-author contact.

**Comment 4**. Response to editor’s comment 20: Please do include Table S1. It is not necessary to include the flowchart, but please do provide the numbers and explanation (e.g. “Of the original 3,273, suicidal ideation data were not available for…” or similar) for those not included in the text of the Methods (approximately at lines 154-155:  “For this study, we analyzed data from a sample of 2,020 participants with at least one measure of suicidal ideation at ages 14, 17, 22, or 28 years.”).
**Response 4.** As suggested, we added the following sentence (lines 155-156):

For this study, we analyzed data from a sample of 2,020 participants with at least one measure of suicidal ideation at ages 14, 17, 22, or 28 years (of the initial 3,273 participants data on suicidal ideation were not available for 1,253).

Table S1 was provided in the supplementary material. Please advise in case you meant to include the Table in the main text.

**Comment 5**. Abstract: Line 31-32: We suggest revising to: “We documented the associations between individual, familial, and environmental factors in childhood with suicidal ideation…”
**Response 5.** This was amended as suggested.

**Comment 6**. Abstract: Line 47: Please clarify if p<0.030 should be p=0.03, or otherwise, please report the exact p value unless p<0.001.
**Response 6.** This p-value is indeed 0.03. This was amended.

**Comment 7**. Author summary: Line 65: Please revise to “To the best of our knowledge, no longitudinal study has been conducted…” or similar.
**Response 7.** This was revised as suggested

**Comment 8**. Author summary: Line 72: Please revise to “2,020” in the second bullet point.
**Response 8.** This was revised

**Comment 9**. Author summary: Line 81-83: We suggest revising these two points slightly to avoid suggesting causal implications (e.g. suggesting a direct link to prevention) of the findings.
**Response 9.** We revised the two points as follows:

• Addressing widespread social and environmental adversities as well as childhood externalizing problems at the population level could potentially be of interest in suicide prevention efforts in South Africa and similar LMIC contexts.

• Considering sex differences may be important to optimize prevention efforts.

**Comment 10.** Author summary: Line 84: Please provide slightly more explanation for this point.
**Response 10.** This point was amended as follows:

• Due attrition, this study was conducted on 62% of the of the initial representative sample. This may influence generalizability of the findings to the initial population.

**Comment 11.** Introduction: Line 87: Please clarify to “...the second or third most common cause of death…” or similar.
**Response 11.** This was clarified as suggested. The sentence now reads as follows:

Among youth aged 15-25 years, suicide ranks as the second or third most common cause of death in most countries [2].

**Comment 12.** Methods: Line 207: Please change the superscript reference to [43] if this is correct.
**Response 12.** This typo was amended

**Comment 13**. Methods: Line 232: Data analysis plan: Please state that there was no formal prospective analysis plan for the study, but please do mention that the analysis protocol was decided upon during study group meetings (please indicate when this took place, i.e. prior to initiation of the analyses), and please indicate that changes to the planned analyses with rationale (e.g. following peer reviewer comments) are described in the Methods.
**Response 13.** As suggested, the following sentences was added (now lines 235-237):

The analysis protocol was decided upon during study group meetings that took place between February and September 2020. However, there was no formal prospective analysis plan for the study. Changes to the initial planned analyses implemented following peer review were documented in the Methods section.

**Comment 14.** Results: Line 264: Please change to P=0.007 if this is accurate. Please report the exact p value, unless p<0.001.
**Response 14.** We amended this typo

**Comment 15.** Results: Line 265 and 266: Please change to “statistically significantly” and “statistically significant” in this sentence. Please also provide the p values for the sex-specific associations reported.
**Response 15.** Changed as suggested

**Comment 16.** Results: Line 279-281: We suggest revising to: “...stratified analyses by sex suggested that the association was stronger among females (OR 1.14, CI 1.05-1.24, P=0.003) than males (OR 1.06, CI 0.95-1.18, P=0.299), although the interaction was not statistically significant…”.
**Response 16.** The sentence was amended as suggested (now lines 285-287).

**Comment 17.** Results: Line 288: Please change to P=0.011 if this is accurate. Please report the exact p value, unless P<0.001.
**Response 17.** This was changed to P=0.011

**Comment 18.** Discussion: Line 294: We suggest that the heading “Main Findings” is not necessarily needed.
**Response 18.** We removed the headings

**Comment 19.** Discussion: Line 299: We suggest revising to: “when rates for males and females were similar…” 
**Response 19.** We revised as suggested

**Comment 20**. Discussion: Lines 402-404: We suggest revising slightly to: “Prevalence rates were higher among females than males, and we found sex differences in the associations of childhood individual, familial, and environmental factors with youth suicidal ideation. As these factors (e.g., externalizing problems, socioeconomic adversity, ACEs) are highly prevalent…”
**Response 20.** We revised the sentence as suggested

**Comment 21**. Page 20: Please remove the sections titled Contributions, Conflict of Interest disclosures, and Data Access from the main text. Please make sure that all information is entered completely and accurately into the relevant sections of the manuscript submission system.
**Response 21.** These sections have now been removed

**Comment 22.** Table 2: Please define RR in the legend.
**Response 22.** This has been defined (line 455)

**Comment 23.** Table 3 and Table 4: Please define OR in the legend.
**Response 23.** This has been defined in both tables as follows:

OR indicates Odds Ratio.

**Comment 24.** Figure 1: Please use a color scheme that does not involve differentiating red and green colors.
**Response 24.** We amended our figure and replaced green with black.

**Comment 25.** Reference 24: Please change the journal title abbreviation to PLoS One.
**Response 25.** This was changed

**Comment 26.** Reference 51: Please change the journal title abbreviation to Lancet.
**Response 26.** This was changed

**Comment 27.** Reference 53: Please update with complete citation information.
**Response 27.** This was updated

**Comment 28.** Table S1 and S2: Please define SD in the legend.
**Response 28.** This was defined

**Comment 29.** Table S3: Please define ACE in the legend.

**Response 29.** This was defined
